# Impacts of Humanized Mouse Models on the Investigation of HIV-1 Infection: Illuminating the Roles of Viral Accessory Proteins *in Vivo*

**DOI:** 10.3390/v7031373

**Published:** 2015-03-23

**Authors:** Eri Yamada, Rokusuke Yoshikawa, Yusuke Nakano, Naoko Misawa, Yoshio Koyanagi, Kei Sato

**Affiliations:** 1 Laboratory of Viral Pathogenesis, Institute for Virus Research, Kyoto University, Kyoto 6068507, Japan; E-Mails: eyamada@virus.kyoto-u.ac.jp (E.Y.); kyusyu.sinden@gmail.com (R.Y.); 111r5134@st.kumamoto-u.ac.jp (Y.N.); nmisawa@virus.kyoto-u.ac.jp (N.M.); ykoyanag@virus.kyoto-u.ac.jp (Y.K.); 2 CREST, Japan Science and Technology Agency, Saitama 3220012, Japan

**Keywords:** HIV-1, accessory protein, humanized mouse model

## Abstract

Human immunodeficiency virus type 1 (HIV-1) encodes four accessory genes: *vif*, *vpu*, *vpr*, and *nef*. Recent investigations using *in vitro* cell culture systems have shed light on the roles of these HIV-1 accessory proteins, Vif, Vpr, Vpu, and Nef, in counteracting, modulating, and evading various cellular factors that are responsible for anti-HIV-1 intrinsic immunity. However, since humans are the exclusive target for HIV-1 infection, conventional animal models are incapable of mimicking the dynamics of HIV-1 infection *in vivo*. Moreover, the effects of HIV-1 accessory proteins on viral infection *in vivo* remain unclear. To elucidate the roles of HIV-1 accessory proteins in the dynamics of viral infection *in vivo*, humanized mouse models, in which the mice are xenotransplanted with human hematopoietic stem cells, has been utilized. This review describes the current knowledge of the roles of HIV-1 accessory proteins in viral infection, replication, and pathogenicity *in vivo*, which are revealed by the studies using humanized mouse models.

## 1. Introduction

**Figure 1:**
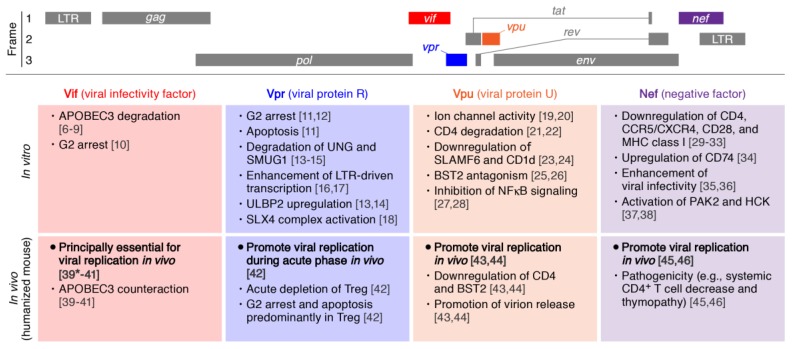
Roles of Human immunodeficiency virus type 1 (HIV-1) accessory proteins *in vitro* and *in vivo*. (Top) The scheme of HIV-1 genome. Three reading frames are respectively indicated. (Middle) Major roles of HIV-1 accessory proteins reported from the experiments using cell cultures. (Bottom) The roles of HIV-1 accessory proteins elucidated from the experiments using humanized mouse models. The numbers in parentheses indicate the references. *, as an exception; Vif is dispensable if a *vif-*deficient CXCR4-tropic HIV-1 (strain LAI) is intravenously inoculated into BLT humanized mice [39]. APOBEC3, apolipoprotein B mRNA editing enzyme, catalytic polypeptide-like 3; UNG, Uracil-DNA glycosylase; SMUG1, single-strand-selective monofunctional uracil-DNA glycosylase 1; LTR, long terminal repeat; ULBP2, UL16 binding protein 2; SLX4, SLX4 structure-specific endonuclease subunit; BST2, bone marrow stromal cell antigen 2; SLAMF6, signaling lymphocyte activation molecule family member 6; PAK2, p21 protein (Cdc42/Rac)-activated kinase 2; HCK, hematopoietic cell kinase.

Human immunodeficiency virus type 1 (HIV-1), a virus belonging to the genus *Retroviridae*, was identified in 1983 as the causative agent of acquired immunodeficiency syndrome (AIDS) [1,2]. HIV-1 genome consists of nine genes (Figure 1, *top*), and five out of the nine genes, *gag*, *pol*, *env*, *tat*, and *rev*, are essential for viral replication [3]. On the other hand, the remaining four genes, *vif*, *vpu*, *vpr*, and *nef*, are not always required for viral replication in *in vitro* studies using cell culture system [3,4]. Recent investigations have shed light on the roles of these viral accessory proteins in counteracting, modulating, and evading various host restriction factors responsible for anti-HIV-1 cellular intrinsic immunity [4,5].

**Figure 2:**
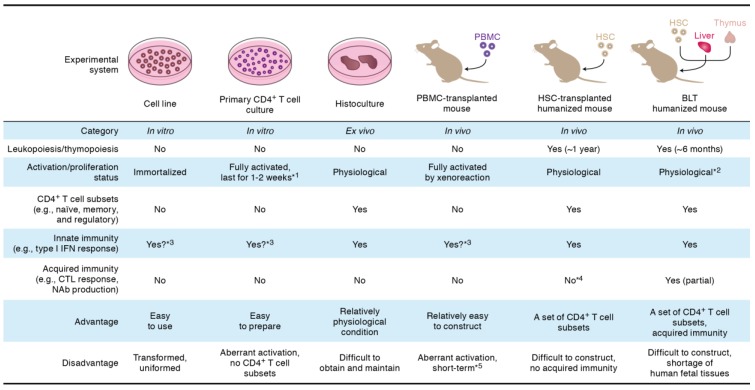
Experimental system for HIV-1 infection. The detailed explanation of each experimental system is described in the text. *^1^, to perform HIV-1 replication assays, primary CD4^+^ T cells should be artificially activated by mitogens (e.g., phytohemaggluttinin and anti-CD3/CD28 antibodies); *^2^, because human thymocytes are efficiently educated in human thymic transplant (*i.e.*, human MHC), the human T cells differentiated in BLT humanized mice may recognize the tissues of recipient mouse as foreign antigen, which can lead to the onset of graft-versus-host reaction; *^3^, these systems are capable of responding type I interferon stimulation, which can lead to the expression of interferon-stimulating genes. However, these systems are incapable of triggering innate immune sensing because of the absence of dendritic cells and macrophages; *^4^, because human thymocytes are educated in the thymus of recipient mouse (*i.e.*, murine MHC), the human T cells differentiated in HSC-transplanted humanized mice are unable to efficiently receive the antigen stimulation from human antigen presenting cells; *^5^, the transplanted human PBMCs recognize the tissues of recipient mouse as foreign antigen and cause graft-versus-host reaction, which results in the aberrant xenoactivation. BLT, bone marrow/liver/thymus; CTL, cytotoxic T lymphocyte; HSC, hematopoietic stem cell; IFN, interferon; NAb, neutralizing antibody; PBMC, peripheral blood mononuclear cell.

For the basic research of HIV-1 infection, *in vitro* cell culture systems including cell lines and primary human CD4^+^ T cells have been extensively utilized (Figure 2). However, the cell lines are transformed and abnormal. Primary human CD4^+^ T cells are artificially activated by mitogens (e.g., phytohemaggluttinin and anti-CD3/CD28 antibodies) to allow efficient HIV-1 replication. On the other hand, only a small portion of cell subsets in CD4^+^ T cells are activated *in vivo*, especially after antigen stimulation [47]. Therefore, the expression patterns of cellular genes, which are positively or negatively associated with HIV-1 replication, may be quite different in *in vitro* and *in vivo*, and it is important to investigate the dynamic interplay between cellular factors and HIV-1 accessory proteins *in vivo*. However, the observations on the roles of HIV-1 accessory proteins, which are summarized in Figure 1, are principally based on the investigations using cell culture system *in vitro*.

To closely mimic HIV-1 infection in *in vivo* conditions, human histoculture systems such as the tissue explants from tonsil [48], cervix [49,50], vagina [49,51], and thymus [52], have been used (Figure 2). Compared to the cell cultures *in vitro*, these *ex vivo* histoculture systems reflect physiological conditions more closely because of the intact tissue architecture with multiple human leukocyte lineages including human CD4^+^ T cell subsets (e.g., naïve, memory, and regulatory cells (Tregs)), monocytes/macrophages, dendritic cells, and stromal cells. However, because of its surgical technique and human donors are needed, it appears to be difficult to routinely use this system for basic HIV-1 research. Moreover, the organ culture can only study HIV-1 infection in the isolated small tissue pieces that might not be ideal for many other experimental purposes.

To reconstruct human immunity *in vivo*, mouse models xenotransplanted with human cells have been developed. One of the classical small animal models is the severe combined immunodeficient (SCID) mouse xenotransplanted with human peripheral blood mononuclear cells (PBMCs) (Figure 2). This PBMC-transplanted mouse model is relatively easy to construct and efficiently allows HIV-1 replication [53]. Also, this model has been used for multiple purposes on HIV-1 research such as the efficacy evaluation of passive immunization of anti-HIV-1 antibodies [54]. However, since the human lymphocytes in this mouse model are aberrantly activated because of xenoreactions against murine antigens [55], the condition of human CD4^+^ T cells are not physiological.

In order to reproduce more physiological condition of human immunity *in vivo*, a new generation mouse model, called “humanized mouse” has been developed [56,57,58,59]. One is the mouse xenotransplanted with human CD34^+^ hematopoietic stem cells (HSCs), while the other, which is called bone marrow/liver/thymus (BLT) mouse, is xenotransplanted with the tissue sections of human fetal thymus and liver as well as human HSCs (Figure 2) [60,61]. Notably, both HSC-transplanted and BLT humanized mouse models are capable of supporting human lymphopoiesis and thymopoiesis for 6–12 months [60,61]. In addition, the human lymphocytes including human CD4^+^ T cell subsets, which are reconstituted in these humanized mouse models, are maintained in a physiological condition [42]. In HSC-transplanted humanized mouse, human acquired immune response (e.g., antibody production and cytotoxic T cell responses) is poorly elicited because human T cells/thymocytes are educated in murine thymus (*i.e.*, the mismatching of major histocompatibility complex [MHC]) (Figure 2) [62,63]. On the other hand, BLT humanized mouse can potently elicit human acquired immunity because human T cells/thymocytes are educated in transplanted human thymic organoid [64,65]. However, it should be noted that BLT humanized mouse may suffer from aberrant immune activation which is triggered by the highly educated human T cells (Figure 2).

In terms of the condition of human CD4^+^ T cells, we can say that these two humanized mouse models are the best choices to reproduce and investigate the dynamics of HIV-1 infection *in vivo* (*i.e.*, under the physiological condition) at present. However, special facilities (e.g., specific pathogen-free condition), surgical technique, and appropriate recipient mice (e.g., NOG and NSG mice; [62,66]) are required for the construction of humanized mouse models. Furthermore, due to the ethical issues in the use and acquisition of the tissues from abortive fetuses for basic investigations, it is difficult to construct BLT humanized mouse model in many countries except for the United States. To circumvent this problem and for better understanding the mechanisms of the development of human immunity, lines of studies to improve the genetic background of the recipient mouse for the establishment of more efficient and appropriate humanized models have been pursued [67,68,69,70].

Because HIV-1 infects and causes disorders only in humans and chimpanzees, there is no perfect animal model to investigate the roles of HIV-1 accessory proteins in viral infection, replication, and pathogenesis *in vivo* so far. In this review, we describe the current state-of-the-art of novel findings on the roles of HIV-1 accessory proteins *in vivo*, which are obtained from the investigations using HSC-transplanted and BLT humanized mouse models.

## 2. Viral Infectivity Factor (Vif)

Apolipoprotein B mRNA editing enzyme, catalytic polypeptide-like 3 (APOBEC3) proteins are cellular cytidine deaminases that convert cytosines in the viral minus-strand cDNA to uracils, which results in the alternation of guanines to adenine in the nascent viral DNA (*i.e.*, G-to-A mutation) [6,7,9]. Human cells encode 7 *APOBEC3* genes, *APOBEC3A, B, C, D, F, G* and *H* [6,8]. Extensive studies using *in vitro* cell cultures have revealed that certain APOBEC3 proteins, particularly APOBEC3D, APOBEC3F, and APOBEC3G, exhibit robust anti-HIV-1 activity principally depending on their enzymatic activity [6,8]. To counteract the anti-viral actions of APOBEC3 proteins, HIV-1 arms its own weapon, Vif. Vif recruits cellular E3 ubiquitin ligase complex, which is composed of cullin 5 (CUL5), elongin B/C (ELOB/C), and core binding factor beta (CBF-β), and degrades APOBEC3 proteins via the ubiquitin/proteasome-dependent pathway (Figure 1) [7]. Moreover, Izumi *et al.* revealed that Vif elicits cell cycle arrest at G2 phase (G2 arrest) independently of its anti-APOBEC3 activity (Figure 1) [10]. To investigate the dynamic interplay between endogenous APOBEC3 proteins and Vif *in vivo*, three previous studies have addressed this issue by using humanized mouse models (Table 1) [39,40,41]. First, Sato *et al.* inoculated CCR5-tropic wild type (WT) HIV-1 (strain JRCSF) and its *vif-*deficient derivative into hHSC-transplanted humanized mice (designated to NOG-hCD34 mice) [40]. Though WT HIV-1 efficiently expanded in humanized mice, *vif-*deficient HIV-1 did not show viremia, strongly suggesting that the replication of *vif-*deficient HIV-1 in humanized mice is canceled by endogenous APOBEC3 proteins expressed in human CD4^+^ T cells of humanized mice. In addition, the accumulation of G-to-A mutations in provirus genome was observed, and notably, lethal mutations (*i.e.*, mutations to stop codons) were preferred. Furthermore, the mRNA expression levels of *APOBEC3* genes in the human CD4^+^ T cells of humanized mice were comparable to those in human peripheral blood (PB) [40]. Therefore, this report suggests that endogenous APOBEC3 proteins expressed in human CD4^+^ T cells can abrogate HIV-1 infection *in vivo* as a result of accumulating G-to-A mutations in proviral DNA, and that Vif counteracts this robust anti-viral activity of endogenous APOBEC3 proteins even *in vivo*.

**Table 1:** HIV-1 mutants used in the studies of humanized mouse models.

*Gene*	Strain	Coreceptor Usage	Mutation Type	Reference ^a^
*vif*	JRCSF	CCR5	Deletion	[40]
	JRCSF	CCR5	Deletion	[39]
	JRCSF	CCR5	Frame shift	[39]
	LAI	CXCR4	Deletion	[39]
	NLCSFV3	CCR5	DRMR/AAAA substitution (4A)	[41]
	NLCSFV3	CCR5	YRHHY/AAAAA substitution (5A)	[41]
	NLCSFV3	CCR5	Both of above (4A5A)	[41]
*vpr*	JRCSF	CCR5	Deletion	[42]
	NL4-3	CXCR4	Deletion	[42]
*vpu*	AD8	CCR5	Deletion	[44]
	NLADA ^b^	CCR5	Deletion	[43]
	NLADA ^b^	CCR5	S52D, S56D substitution	[43]
*nef*	LAI	CXCR4	Deletion	[46]
	LAI	CXCR4	Frame shift	[45]
	LAI	CXCR4	*fs*Δ-1 ^c^	[45]
	LAI	CXCR4	*fs*Δ-13 ^c^	[45]
	LAI	CXCR4	P72A, P75A substitution	[45]

^a^ References, which corresponds to those in Figure 1, are shown; ^b^ The virus used in this study contains GFP reporter via internal ribosome entry site; ^c^ Reverted *nef* ORFs, which are obtained in the mice infected with HIV-1 carrying a frame shift mutation in *nef*.

Second, Krisko *et al.* inoculated WT and certain kinds of *vif* mutant HIV-1 into BLT humanized mice [39]. Similar to the previous report [40], *vif-*deficient CCR5-tropic HIV-1 (strain JRCSF) was unable to propagate in BLT mice [39]. On the other hand, 6 out of the 16 BLT mice intravenously inoculated with the virus carrying a frame shift mutation in *vif* (HIV-1 *vi*fFS, strain JRCSF) exhibited viremia. Since *vif* open reading frame (ORF) is restored in the six mice displayed viremia, these results further suggest that Vif is prerequisite for viral spread *in vivo* to counteract APOBEC3-mediated anti-viral effect*.*

When CCR5-tropic HIV-1 *vif*FS was directly injected into the spleen, liver, lung, or human thymic organoid of BLT mice, only the mice injected the virus solution into human thymic organoid exhibited systemic viremia with the reversion of *vif* ORF [39]. Moreover, the authors revealed that the mRNA expression levels of *APOBEC3F* and *APOBEC3G* in the human thymocytes of humans and BLT mice was significantly lower than those in the human CD4^+^ T cells in peripheral tissues [39]. Therefore, these findings suggest that thymocytes can allow the partial replication of CCR5-tropic HIV-1 *vif*FS and its *vif* restoration, which leads to the systemic spread of the restored viruses. These further suggest that CCR5-tropic HIV-1 is unable to exhibit systemic infection without Vif regardless of infection route.

In contrast to the observations in CCR5-tropic HIV-1-infected BLT mice, it was surprising that the BLT mice intravenously inoculated with CXCR4-tropic *vif-*deficient HIV-1 (strain LAI) showed a prolonged (until 14 weeks postinfection [wpi]) viremia [39]. More importantly, this virus spread occurred without *vif* restoration, suggesting that Vif is dispensable for the replication of CXCR4-tropic *vif-*deficient HIV-1 in BLT mice. In this regard, CCR5 is limitedly expressed in the thymus, whereas 30%–40% of thymocytes express CXCR4 [71,72,73]. Since human thymocytes express low levels of *APOBEC3F* and *APOBEC3G*, as mentioned above, human thymus can be susceptible to CXCR4-tropic *vif-*deficient HIV-1 propagation. However, it should be noted that the majority of HIV-1 isolated from patients is CCR5-tropic, while CXCR4-tropic HIV-1 can infrequently emerge during the onset of AIDS [74,75]. Since the replication of CCR5-tropic *vif-*deficient HIV-1 infection in BLT mouse needs the restoration of *vif* and is achieved only via artificial infection route (*i.e.*, direct injection into the human thymic organoid, which is a unique organ in BLT mouse), it would be infeasible for CCR5-tropic HIV-1 to propagate *in vivo* via relatively natural infection routes (e.g., intrarectal, intravaginal, or intravenous infections). Further, the observations in CXCR4-tropic *vif-*deficient HIV-1-infected BLT mice may occur only the late stage of HIV-1 infection in patients.

Third, Sato *et al.* have recently utilized three kinds of site-directed Vif mutants: DRMR/AAAA (4A), YRHHY/AAAAA (5A), double mutant (4A5A), respectively [41]. Vif interacts with APOBEC3D and APOBEC3F via ^14^DRMR^17^ motif, while interacts with APOBEC3G via ^4^°YRHHY^44^ motif [76,77]. Hence, 4A HIV-1 is susceptible only to APOBEC3D and APOBEC3F, while 5A HIV-1 is susceptible only to APOBEC3G. By using these CCR5-tropic viruses (strain NLCSFV3) and NOG-hCD34 humanized mouse model, the authors demonstrated that endogenous APOBEC3D, APOBEC3F, and APOBEC3G exert strong anti-HIV-1 activity *in vivo* [41]. In addition, the growth kinetics of 4A HIV-1 negatively correlated with the expression level of *APOBEC3F* but not of *APOBEC3D*, suggesting that endogenous APOBEC3F more critically modulates 4A HIV-1 replication *in vivo* than APOBEC3D. It was of particularly noteworthy that the viral RNA in the plasma of 4A HIV-1-infected mice was significantly diversified compared to those of WT, 5A, and 4A5A HIV-1-infected mice [41]. Furthermore, a mutated virus (E25K mutation in the V3 region of envelope glycoprotein), which is capable of using both CCR5 and CXCR4 as entry coreceptor, has specifically emerged in 4A HIV-1-infected mice [41]. Altogether, these findings suggest that endogenous APOBEC3D, APOBEC3F, and APOBEC3G fundamentally are intrinsic restriction factors against HIV-1 *in vivo*, but, at the same time, that APOBEC3D and APOBEC3F are capable of promoting viral diversification and evolution *in vivo*. This is the first report *in vivo* demonstrating that endogenous APOBEC3D and APOBEC3F potently promote viral diversification and evolution, which can be beneficial for viruses (e.g., emergence of quasispecies resistant to anti-HIV-1 drugs anti-viral immunity).

## 3. Viral Protein R (Vpr)

Vpr is a small (96 amino acids) protein, but potentially possesses multiple biological functions. Major roles of Vpr *in vitro* are G2 arrest and apoptosis (Figure 1) [11,12]. Although the mechanisms of action of Vpr leading to G2 arrest and apoptosis remain controversial [11], a paper has recently suggested that Vpr induces the activation of the cellular structure-specific endonuclease regulator SLX4 complex, which results in G2 arrest (Figure 1) [18]. Also, Vpr has the potential to enhance HIV-1 long terminal repeat-driven transcription [16,17]. In the field of immunology, Vpr expressed in the infected cells upregulates UL16 binding protein 2 (ULBP2; also known as NKG2D ligand), a counter receptor for natural killer cell-specific receptor, which leads to natural killer cell-mediated killing [78,79]. Moreover, Vpr recruits a cellular E3 ubiquitin ligase complex, which is composed of cullin 4 (CUL4), damage-specific DNA binding protein 1 (DDB1), and Vpr binding protein (VPRBP), and degrades some cellular proteins such as Uracil-DNA glycosylase (UNG; also known as UNG2) [13,14] and single-strand-selective monofunctional uracil-DNA glycosylase 1 (SMUG1) (Figure 1) [15]. When compared to the observations in Vif (described above) and Vpu (described below), however, the virological significance of Vpr-mediated ubiquitin ligase complex still remains unsolved.

To address the role of Vpr in HIV-1 infection *in vivo* and its contribution to disease development, Sato *et al.* inoculated CCR5-tropic *vpr*-deficient HIV-1 (strain JRCSF) into NOG-hCD34 humanized mice (Table 1) [42]. CCR5-tropic *vpr*-deficient HIV-1-infected mice showed a significantly lower level of viremia during the acute phase of infection (4 and 7 days postinfection [dpi]) compared with WT HIV-1 [42]. In addition, the level of infected Tregs in *vpr*-deficient HIV-1-infected mice was significantly lower than that in WT HIV-1-infected mice [42]. Moreover, WT but not *vpr*-deficient CCR5-tropic HIV-1-infected mice displayed the acute depletion of Tregs in PB and spleen [42]. Furthermore, Vpr-dependent G2 cell cycle arrest and apoptosis are predominantly observed in infected Tregs [42]. Importantly, these were observed in the mice infected with CCR5-tropic HIV-1 (strain JRCSF), whereas there were no significant differences in the case of CXCR4-tropic HIV-1 (strain NL4-3) [42]. These findings suggest that the Vpr-dependent Treg depletion is dependent on viral coreceptor usage. In this regard, Tregs are highly susceptible to CCR5-tropic HIV-1 infection because the CCR5 expression levels on Tregs are higher than those on naive and memory CD4^+^ T cells [42,80]. Also, Tregs are more susceptible to Vpr-mediated G2 arrest and apoptosis because they are actively proliferating. On the other hand, CXCR4 is broadly expressed on all CD4^+^ T cell subsets [42,80,81]. Therefore, the Vpr-dependent G2 arrest and apoptosis can be preferentially triggered by CCR5-tropic but not CXCR4-tropic HIV-1 *in vivo*.

It is known that Treg plays a crucial role in the maintenance of immune homeostasis [82]. In CCR5-tropic HIV-1-infected mice, Vpr-dependent depletion of Treg resulted in immune activation [42], which is a hallmark in the patients infected with HIV-1 [83]. Altogether, these findings suggest that Vpr enhances CCR5-tropic but not CXCR4-tropic HIV-1 replication mediating G2 arrest and apoptosis *in vivo* by exploiting Treg during the acute phase of infection. This Vpr-dependent Treg depletion may lead to immune activation and provide a pool of activated CD4^+^ T cells, which supports subsequent HIV-1 expansion *in vivo*.

## 4. Viral Protein U (Vpu)

Vpu is a transmembrane protein and has been classically recognized as a “viroporin”, which works as ion channel (Figure 1) [19,20]. In addition, Vpu degrades some host proteins such as CD4 molecule, the receptor for HIV-1 entry, through the ubiquitin/proteasome pathway [84]. Vpu also downregulates signaling lymphocyte activation molecule family member 6 (SLAMF6; also called NTB-A), a transmembrane protein potently inducing natural killer cell-mediated killing [24] and CD1d molecule [23] from the cell surface of infected cells (Figure 1).

It was known that certain human CD4^+^ T cell lines (e.g., Jurkat cells), primary CD4^+^ T cells, monocyte-derived macrophages, and HeLa cells are incapable of producing the *vpu*-deficient HIV-1 virions [21,22]. Neil *et al.* and Van Damme *et al.* identified that the cellular factor, bone marrow stromal cell antigen 2 (BST2; also known as CD317, HM1.24 and tetherin) [25,26]. BST2 is an interferon-stimulated protein and is endogenously expressed on human CD4^+^ T cells and macrophages [85,86]. On the other hand, Vpu downregulates BST2 from the cell surface and counteracts BST2-mediated anti-viral activity (Figure 1) [26,86]. The Vpu-mediated BST2 downregulation is dependent on β-transducin repeat-containing protein 1 (BTRC; also called β-TrCP1), an E3 ubiquitin ligase, similar to the manner by which CD4 is downregulated [84,87,88,89,90]. Moreover, it has been recently revealed that Vpu inhibits the activation of NFκB signaling [27,28]. These observations were brought from *in vitro* studies using cell culture systems, however, the role of Vpu in HIV-1 replication *in vivo*, particularly its antagonism of BST2 *in vivo*, remains unresolved.

Sato *et al.* [44] and Dave *et al.* [43] investigated the role of Vpu in HIV-1 expansion *in vivo* using humanized mouse models (Table 1). In the former paper, NOG-hCD34 humanized mice were inoculated with WT or *vpu*-deficient HIV-1 (strain AD8) at a relatively high dose (300,000 TCID_50_) [44]. The authors revealed that the viral load of *vpu*-deficient HIV-1 was 8.5-fold lower than in that of WT HIV-1 at 7 dpi, suggesting that *vpu*-deficient HIV-1 more slowly propagates in humanized mice than WT HIV-1 during the initial phase of infection [44]. At 7 dpi, although the percentage of Gag-positive cells (*i.e.*, virus-producing cells) in the spleen of *vpu*-deficient HIV-1-infected mice was similar to that of WT HIV-1-infected mice, it was of particularly noteworthy that the amount of cell-free virions in the spleen of *vpu*-deficient HIV-1-infected mice was quite lower (61.8-fold) than in that of WT HIV-1-infected mice [44]. Moreover, the authors revealed that the expression levels of BST2 and CD4, but not SLAMF6, on the surface of Gag-positive cells in the spleen of WT HIV-1-infected mice were significantly lower than in those of *vpu*-deficient HIV-1 at 7 dpi [44]. These findings suggest that Vpu downregulates BST2 and CD4 from the surface of virus-producing cells to promote the release of nascent virions, which augments the initial burst of HIV-1 replication *in vivo*.

In the latter paper [43], hHSC-transplanted humanized mice were infected with WT HIV-1 or *vpu*-deficient HIV-1 (strain NLADA) at a relatively low dose (5,000 TCID_50_). Until 14 wpi, the viral load in the plasma of *vpu*-deficient HIV-1-infected mice was ~5-150-fold lower than that of WT HIV-1-infected mice until 14 wpi [43]. Similar to the former paper [44], BST2 downregulation was detected on the surface of Gag-positive cells in WT HIV-1 infected mice. On the other hand, in *vpu*-deficient HIV-1-infected mice, the expression level of surface BST2 on Gag-positive cells was higher than that of Gag-negative cells [43]. Such BST2 upregulation on the uninfected cells was reported in the former paper during the chronic phase of infection (21 dpi) [44] and the individuals infected HIV-1 [91]. Because BST2 is an interferon-stimulated gene, the BST2 upregulation can be triggered by type I interferon, which is induced by HIV-1 infection. Taken together, these findings suggest that Vpu downregulates surface BST2 *in vivo* for the promotion of viral production and propagation regardless of input viral dose.

To better understand the association of BTRC with the role of Vpu, Dave *et al.* [43] used a mutant virus, HIV-1 Vpu^S52D/S56D^. This Vpu is incapable of recruiting BTRC and thereby is unable to degrade BST2. The authors inoculated WT HIV-1, *vpu*-deficient HIV-1, or HIV-1 Vpu^S52D/S56D^ into humanized mice at a relatively high dose (~500,000 TCID_50_) [43]. At 21 dpi, although *vpu*-deficient HIV-1-infected mice exhibited severely lower viremia (~15-fold) compared to WT HIV-1-infected mice, the decrease in the viral load of HIV-1 Vpu^S52D/S56D^ comparing to WT HIV-1 was relatively mild (~3-fold) [43]. Moreover, HIV-1 Vpu^S52D/S56D^ partially downregulated surface BST2 (20%–30%) on Gag-positive cells of infected mice, whereas *vpu*-deficient HIV-1 was unable to downregulate BST2 on the surface of Gag-positive cells [43]. Since WT HIV-1 strongly downregulated surface BST2 (50%–60%) compared to *vpu*-deficient HIV-1 and HIV-1 Vpu^S52D/S56D^, these findings suggest that the efficacy of BST2 downregulation associates with the level of viral spread *in vivo*. However, it should be considered that the mutations of these serine residues in Vpu also abrogates downmodulation of CD4 [92] and inhibition of NFκB signaling [28]. Therefore, the observations in the humanized mice infected with HIV-1 Vpu^S52D/S56D^ [43] may not be solely ascribed to the lack of tetherin counteraction.

Notably, Sato *et al.* [44] further revealed that the efficacy of *vpu*-deficient HIV-1 infection in humanized mice was significantly lower than that of WT HIV-1. This observation suggests that Vpu potently enhances the efficacy of infection in humans, which leads to the promotion of human-to-human HIV-1 transmission. It is of interest that Vpu proteins of pandemic HIV-1 (group M) possess anti-BST2 ability, while those of non-pandemic HIV-1 (groups N, O, and P) do not or less [2,4,93,94,95]. Therefore, the worldwide epidemic of HIV-1 group M might be attributed to the Vpu-mediated BST2 antagonism to some extent.

## 5. Negative Factor (Nef)

*In vitro* investigations have revealed that Nef is a pluripotent protein. For instance, Nef downregulates CD4 [30,35], MHC class I [30,32], CCR5/CXCR4 coreceptors [29,30,31], and CD28 [33] from the surface of infected cells (Figure 1). On the other hand, Nef upregulates CD74, the invariant chain of MHC class II [34]. In addition, Nef potently enhances the infectivity of released virions [35,36], though the molecular mechanism of this action remains unclear. Moreover, Nef induces the activation of cellular protein kinases such as p21 protein (Cdc42/Rac)-activated kinase 2 (PAK2) and a tyrosine kinase, hematopoietic cell kinase (HCK) (Figure 1) [37,38].

*In vitro* studies using activated human primary CD4^+^ T cells and *ex vivo* studies using human tonsil tissue cultures have suggested that CD4 downregulation is more critical for Nef to promote viral replication than MHC class I downregulation [96,97,98]. Importantly, the patients infected with *nef*-defective HIV-1 failed to develop AIDS [99,100,101,102,103,104]. Given that a long-term non-progressor, who has been infected with attenuate *nef*-defective viruses, exhibited acute CD4 T cell decrease in PB through the superinfection of *nef*-proficient virus [105], these notions strongly suggest that Nef closely associates with viral pathogenesis and disease progression *in vivo*.

To directly investigate the roles of Nef in HIV-1 replication and pathogenesis *in vivo*, a BLT humanized mouse model and *nef-*deficient HIV-1 (strain LAI) were used (Table 1) [46]. Although WT HIV-1-infected mice exhibited a profound loss of human CD4^+^ T cells and thymocytes, *nef-*deficient HIV-1-infected mice showed neither CD4^+^ T cell decrease nor thymopathy [46]. Also, the growth of *nef-*deficient HIV-1 was clearly lower than that of WT HIV-1 [46], suggesting that Nef is necessary for elevated viral replication *in vivo*, which results in the depletion of thymocytes.

Another paper examined whether *nef*-defective virus recovers Nef function by using HIV-1 (strain LAI) with a frame shift mutation in *nef* (HIV-1 *nef*FS) and a BLT humanized mouse model (Table 1) [45]. Watkins *et al.* detected two restored *nef* ORFs, which are designated to *fs*Δ-1 and *fs*Δ-13 respectively, in the PB of BLT humanized mice infected with HIV-1 *nef*FS at 8 wpi [45]. *In vitro* assays revealed that MHC class I but not CD4 molecule can be downregulated by *fs*Δ-1 and *fs*Δ-13, and that the infectivity and replication kinetics of the viruses possessing these two mutated Nef proteins were comparable to that of parental HIV-1 [45]. However, in BLT mice, the growth of HIV-1 *fs*Δ-1 and HIV-1 *fs*Δ-13 was ~3-fold lower than WT HIV-1, and the level of systemic CD4^+^ T cell decrease and thymopathy by HIV-1 *fs*Δ-1 and HIV-1 *fs*Δ-13 infections (~50% reduction) were milder than WT HIV-1 (~90% reduction) [45]. These results suggest the importance of CD4 downregulation by Nef for efficient viral growth and pathogenicity.

To explore the roles of Nef other than CD4 downregulation in viral replication kinetics *in vivo* and pathogenesis, Watkins *et al.* used the other mutant, HIV-1 Nef^P72A/P75A^ [45]. This Nef has mutations in the highly conserved SH3 binding domain (^72^PQVPLR^77^), and thereby, is unable to interact with several cellular proteins such as PAK2 and HCK [37,106,107]. Yet, Nef^P72A/P75A^ is capable of downregulating CD4 molecules [107,108]. In BLT humanized mice, HIV-1 Nef^P72A/P75A^ efficiently expanded comparable to WT HIV-1, and the level of systemic CD4^+^ T cell decease by HIV-1 Nef^P72A/P75A^ infection was similar to WT HIV-1 [45]. Altogether, these findings suggest that the *in vivo* phenotype of Nef is highly dependent on its ability to downregulate CD4 molecules but minimally on the interaction with cellular proteins via SH3 domain. However, it should be noted that these two previous studies focusing on the roles on Nef *in vivo* [45,46] were performed by using CXCR4-tropic HIV-1 (strain LAI) (Table 1). It is known that CCR5-tropic viruses are predominant in patients, while CXCR4-tropic viruses occasionally emerge at the end stage of HIV-1 infection [74,75]. Therefore, it would be important to assess the *in vivo* phenotype of Nef not only by CXCR4-tropic HIV-1 but also by using CCR5-tropic HIV-1 and humanized mouse models.

## 6. Future Perspective

Here we summarized the current knowledge of the roles of HIV-1 accessory proteins in viral replication and pathogenesis in humanized mouse models*.* As summarized in Figure 1, there are some overlaps and discrepancies in the observations between *in vitro* and *in vivo*. This indicates that certain findings in *in vitro* studies may not reflect the *bona fide* roles of HIV-1 accessory proteins in viral infection, replication, and pathogenesis *in vivo*, and further suggests that the findings brought from *in vitro* experiments should be verified by *in vivo* experiments using humanized mouse models. Recently, humanized mouse models have been utilized for the evaluation of anti-HIV-1 prevention/therapeutic strategies *in vivo* [109,110]. Moreover, these animal models are unique and robust experimental models to elucidate the “authentic” functions of HIV-1 proteins in the dynamics of viral infection *in vivo*, which can lead to the detailed knowledge of HIV-1 infection and the development of novel anti-viral strategies. Furthermore, it should be noted that laboratory-adapted molecular clones of HIV-1 (e.g., strains LAI, AD8, NL4-3, and their derivatives) have been used in most of humanized mouse studies so far (Table 1). However, recent studies have shown that especially accessory proteins are often not fully functional in these laboratory-adapted strains since they are often dispensable for replication *in vitro* [111,112]. This also raises a possibility that the roles of accessory proteins, which have been brought from the studies using laboratory-adapted molecular clones, may be underestimated. Therefore, we highlight the importance of analyzing clones of primary isolates (e.g., transmitted/founder and chronic viruses) to investigate the *bona fide* roles of Vpr, Vif, Vpu and Nef *in vivo*. Future basic scientific investigations focusing on the host-virus interaction including the roles of HIV-1 accessory proteins, using humanized mouse models will shed light on the not-yet-identified but crucial aspects of the dynamics of HIV-1 infection.
